# Effect of lacosamide in peripheral neuropathic pain: study protocol for a randomized, placebo-controlled, phenotype-stratified trial

**DOI:** 10.1186/s13063-019-3695-7

**Published:** 2019-10-11

**Authors:** Malin E. Carmland, Melissa Kreutzfeldt, Jakob V. Holbech, Niels T. Andersen, Troels S. Jensen, Flemming W. Bach, Søren H. Sindrup, Nanna B. Finnerup

**Affiliations:** 1 Danish Pain Research Center, Department of Clinical Medicine, Aarhus University, Palle Juul-Jensens Boulevaard 99, DK-8200 Aarhus N, Denmark; 2 Department of Neurology, Aarhus University Hospital, Aarhus, Denmark; 3 Department of Neurology, Odense University Hospital, Odense, Denmark; 4 Department of Public Health, Aarhus University, Aarhus, Denmark

**Keywords:** Neuropathic pain, Precision medicine, Lacosamide, Randomized controlled trial

## Abstract

**Background:**

Neuropathic pain is a common pain condition that has a major negative impact on health-related quality of life. However, despite decades of research, it remains difficult to treat neuropathic pain. Lacosamide is a sodium-channel blocker that is efficacious in animal models of neuropathic pain. In humans, its effect in neuropathic pain is inconclusive, based on inconsistent results and very large placebo responses. Previous trials have not used patient stratification or looked for predictors for response.

**Methods:**

This study will be conducted as a multicenter, randomized, double-blind, placebo-controlled, parallel, phase 2, proof-of-concept, phenotype-stratified study. The study will enroll 108 patients with peripheral neuropathic pain who will be randomized to a 12-week treatment with lacosamide or placebo up to 400 mg/day in a 2:1 ratio. The primary objective is to compare the change in the mean value of the patients’ daily ratings of average pain intensity from baseline to the last week of treatment in patients with and without the irritable nociceptor phenotype in the per-protocol population. A supportive objective is to compare the effect of lacosamide with that of placebo in the two phenotypes. Secondary and tertiary outcomes include the Patient Global Impression of Change, pain relief, presence of 30% and 50% pain reduction, sleep disturbance, depression, and anxiety.

**Discussion:**

We will examine the concept of individualized therapy based on phenotyping, and expect that this study will provide important information on the usefulness of lacosamide in the treatment of peripheral neuropathic pain.

**Trial registration:**

ClinicalTrials.gov, NCT03777956. Registered on 18 December 2018.

**Electronic supplementary material:**

The online version of this article (10.1186/s13063-019-3695-7) contains supplementary material, which is available to authorized users.

## Background

Neuropathic pain is a common pain condition that has a major negative impact on health-related quality of life [1]. Despite many years of intensive research into the prevention and management of this pain condition, it remains difficult to treat. Evidence-based recommendations list three drug classes as first-line therapies: tricyclic antidepressants (TCAs), α2δ calcium channel ligands (gabapentin and pregabalin), and serotonin and noradrenaline re-uptake inhibitors (SNRIs) (duloxetine, and venlafaxine) [2]. However, many patients are left with no or limited pain relief using these drugs in maximum tolerated doses or drug combinations. There is therefore an urgent need for other drugs for treating neuropathic pain. There is increasing interest in identifying predictive biomarkers associated with a specific tractable pain mechanism linked to a drug with a known mechanism of action [3, 4]. One promising approach is to use quantitative sensory testing (QST), which involves standardized mechanical and thermal stimuli to assess loss (negative signs) and gain (positive signs) of sensory function. In a phenotype-stratified randomized, double-blind, placebo-controlled study, our research group has recently shown that the sodium channel blocker oxcarbazepine reduced pain more in patients with the so-called irritable nociceptor phenotype than in patients without this phenotype [5]. In addition, a malfunctioning descending pain modulation (e.g. assessed by the conditioned pain modulation (CPM) test) [6] and patient-reported outcomes assessed with validated questionnaires such as the Neuropathic Pain Symptom Inventory (NPSI) may represent predictive biomarkers for drug efficacy [7].

Lacosamide is a functionalized amino acid molecule, developed as an antiepileptic drug. It selectively enhances the slow inactivation of voltage-gated sodium channels and interacts with the collapsin-response mediator protein-2 (CRMP-2), which is involved in neurotrophic pathways [8]. Lacosamide is efficacious in animal models of neuropathic pain [9]. In humans, the effect of lacosamide on neuropathic pain is inconclusive, based on inconsistent results and very large placebo responses (i.e. a large reduction in pain intensity during placebo treatment) in the few randomized controlled trials (RCTs) [2, 10–13]. Improvements were seen for secondary variables such as sleep, patient global impression of change (PGIC), quality of life, and pain interference; and two trials with multiple dosing showed efficacy of lacosamide 400 mg on the primary outcome [11, 12]. Recently, a study in patients with SCNA9A-associated painful small-fiber polyneuropathy has been carried out [14]. These studies did not reveal serious or clinically relevant safety issues, and the majority of reported adverse events (most commonly dizziness, nausea, and headache) were mild to moderate. Previous lacosamide trials have not used patient stratification or looked for predictors. The aim of this study is therefore to assess whether specific pain phenotypes based on sensory testing are linked to an increased response to lacosamide in patients with peripheral neuropathic pain.

**Fig. 1:**
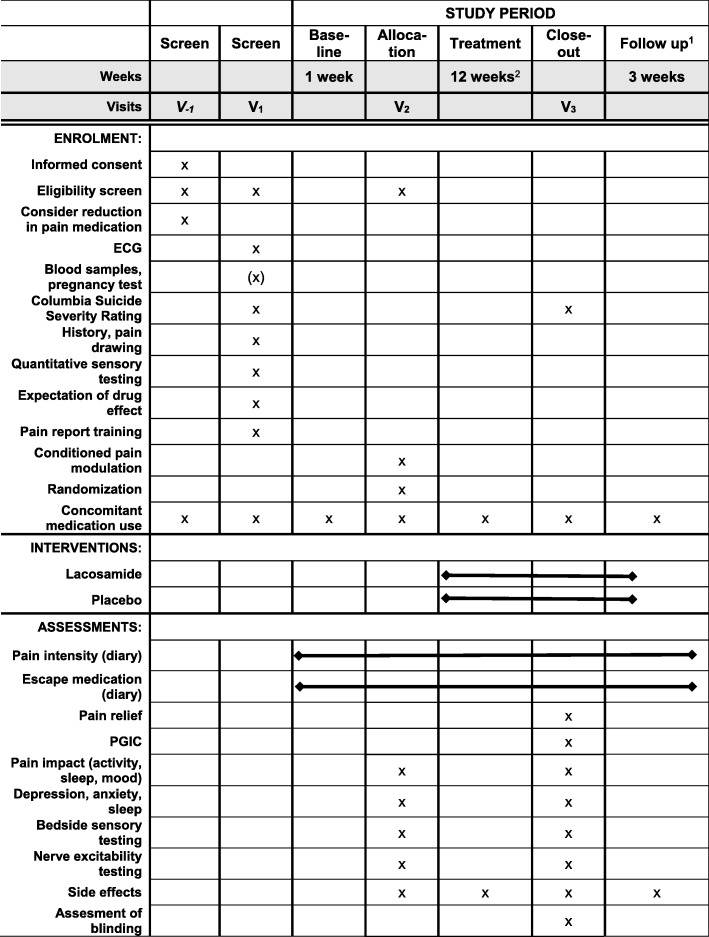
Standard Protocol Items: Recommendations for Interventional Trials (SPIRIT) figure. Schedule of enrolment, interventions, and assessment. ^1^The 3-week follow-up includes a 1-week tapering down and a 2-week follow-up with no medication. Telephone call after the 3-week follow-up. In the case of unresolved side effects at this call, an additional call is scheduled after 1–4 weeks. ^2^Patients treated with pregabalin will come for an extra visit. ECG electrocardiogram, PGIC Patient Global Impression of Change

Standard Protocol Items: Recommendations for Interventional Trials (SPIRIT) were used in writing this manuscript (see Fig. 1 and Additional file 1).

## Methods/design

### Objective

**Fig. 2:**
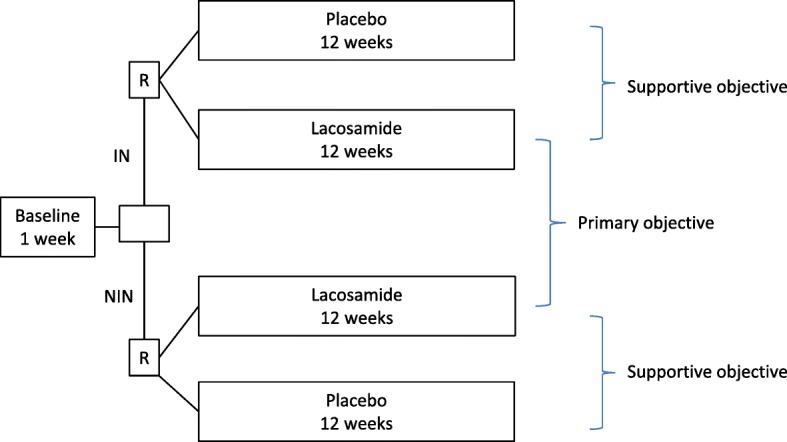
Primary and supportive objectives. IN irritable nociceptor, NIN non-irritable nociceptor, R randomization

The primary objective is to compare the change in average intensity of neuropathic pain from the baseline week to the last week of lacosamide treatment in patients with and without the irritable nociceptor phenotype who complete at least 2 weeks on stable medication with at least 100 mg bid; that is, in the per-protocol (PP) population (Fig. 2).

The supportive objective is to compare the effect of lacosamide with placebo in the two phenotype groups in the PP population (Fig. 2). Although we do not expect a phenotype difference in the response to placebo, a comparison of the effect of lacosamide versus placebo is needed to justify that the phenotype is a predictive biomarker for the effect of lacosamide.

### Exploratory objectives

- To analyze whether the change in pain intensity from baseline to the last week of lacosamide and placebo treatment depends on preserved thermal sensation (QST), gain of sensation (QST), pain characteristics as determined by the NPSI, or evoked pain ratings in the bedside sensory testing
- If there is no phenotype difference, we will compare the primary and secondary outcomes in the total population
- To analyze whether there is a correlation between the percentage change in pain score from the baseline week to the last week of treatment and the effect of conditioned pain modulation (CPM) [6]
- To analyze predictors of the placebo response (CPM, patient expectation, age, gender, anxiety and depression scores at first examination, pain duration, baseline pain variability, and adverse events)

### Study design/plan

**Fig. 3:**
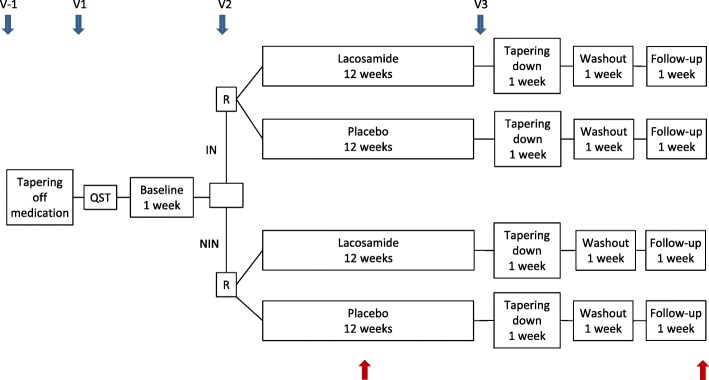
Study design. Blue arrows, visits; red arrows, telephone calls. IN irritable nociceptor, NIN non-irritable nociceptor, QST quantitative sensory testing, R randomization, V visit

The study will be conducted as a multicenter, randomized, double-blind, placebo-controlled, parallel, phase 2, proof-of-concept study, a collaboration between the Department of Neurology, Odense University Hospital, the Danish Pain Research Center, Aarhus University, and the Department of Neurology, Aarhus University Hospital. The study comprises a 1-week baseline period and a 12-week treatment period followed by a 3-week period including a tapering down and follow-up period (Fig. 3). Patients should have an average intensity of pain of at least 4 (NRS scale) and not above 9 in the baseline period, but this is not revealed to the patient. Before inclusion, the diagnosis of probable or confirmed neuropathic pain will be confirmed by the investigator using a detailed pain history, focused clinical and neurological examination, and evaluation of previous paraclinical examinations.

At screening, QST will be performed according to the standard practice and a standard protocol [15]. QST will be used to categorize patients as having either the irritable or the non-irritable nociceptor phenotype [5]. QST will be done by a nurse not otherwise involved in the study, and the investigators and study nurses involved in the study will be blinded to the results of the QST and the phenotype of the patients. The patients are not informed about their pain phenotype and its expected impact on pain relief, and thus the exact definition of irritable nociceptor phenotype is provided only in the study protocol submitted to the ethical committee. Trial participants and all personnel involved in the study are blinded to assignment to interventions.

At the screening, patients will be trained in reporting pain intensity accurately, using cases to improve the patients’ understanding of the numeric rating scale (NRS) and how to score their average daily pain.

### Study drugs

The trial medication will be supplied from the pharmacy at Odense University Hospital (Sygehus Apotek Fyn). Lacosamide (50 mg) and identical placebo are given as capsules and taken orally twice a day. We have adapted a slow titration in an attempt to increase tolerability and reduce dropout. Patient will start with 50 mg bid followed by a 6-week titration phase, increasing the dose by 50 mg weekly to 150 mg bid in week 5 with an increase to 200 mg bid in week 6. The maximal dose is 400 mg/day (200 mg bid), and the dose is kept constant from week 6 until the end of the treatment period. If patients experience intolerable side effects, they are permitted to lower the dose to the highest tolerable dose, but not lower than 100 mg bid. After the treatment, the patients will have a 1-week tapering-down period, following which they will continue in the study for another 2 weeks. This is done to assess whether the pain returns to baseline values, which is relevant in order to understand possible placebo responses. The allowed escape medicine for any type of pain during all of the study periods is paracetamol, up to 4000 mg daily, and the intake is noted in a diary.

### Study population

Patients with peripheral neuropathic pain will be recruited from the Department of Neurology, Odense University Hospital, the Pain and Headache Clinic, Department of Neurology, Aarhus University Hospital, other departments, and via advertisements. Patients with peripheral neuropathic pain following peripheral nerve injury (including amputation), painful polyneuropathy, postherpetic neuralgia, and painful radiculopathy will be included. Patients with central neuropathic pain (e.g. pain due to stroke, multiple sclerosis, and spinal cord injury) will not be included. Patients with trigeminal neuralgia, which is sometimes partly a central neuropathic pain and has different treatment recommendations, will also not be included. Patients with CRPS type I or II will not be included.

#### Inclusion criteria

1. Age ≥ 18 years
2. Verified probable or definite peripheral neuropathic pain for at least 3 months [16]
3. Average pain intensity of at least 4 and not above 9 on an 11-point (0–10) NRS during the 7-day baseline week
4. Written informed consent (Additional file 2)

#### Exclusion criteria

1. Other causes of pain in the same area or other concomitant pain that cannot be distinguished from the neuropathic pain
2. Patients who cannot cooperate or are expected not to be able to complete the project and patients who do not speak Danish
3. Known and current cardiac conduction disturbance (2° or 3° atrioventricular (AV) block, prolonged QTc interval > 450 ms, heart rate < 50 or > 110 bpm, QRS interval > 120 ms (ECG required)), significant cardiac, renal, or liver disease, or other severe illness; sitting diastolic blood pressure below 50 mmHg or above 105 mmHg; in patients treated with pregabalin, also PQ interval > 0.2 s
4. Major depressive episode within 6 months, recurrent depressive disorder or other significant psychiatric disease, and alcohol, illicit drug or drug abuse
5. Pregnancy or lactation
6. Woman of childbearing potential, unless they use acceptable effective contraception as defined in the Clinical Trials Facilitation Group (CTFG) during the study and at least 2 weeks after, or if their male partner has had a vasectomy and is their sole partner; a negative pregnancy test is required; acceptable effective contraception is defined in the CTFG (http://www.hma.eu/fileadmin/dateien/Human_Medicines/01-About_HMA/Working_Groups/CTFG/2014_09_HMA_CTFG_Contraception.pdf)
7. Known allergy to lacosamide or excipients
8. Concomitant pain treatment with tricyclic antidepressants, topical analgesics (lidocaine, capsaicin), lamotrigine, oxcarbazepine, cannabinoids, or strong opioids that cannot be discontinued; other treatments for neuropathic pain are allowed in a stable dose (from 14 days before randomization to completion of the trial) if they cannot be tapered off completely
9. Concomitant treatment with products known to be associated with PQ (PR) prolongation other than pregabalin
10. Patients inappropriate for placebo treatment
11. Planned surgery
12. Use of sodium channel blockers within at least five half-lives and investigational drugs within 30 days
13. Patients on a controlled sodium diet unless the amount of sodium in the capsules is acceptable for their diet
14. The score “yes” on item 4 or item 5 of the Suicidal Ideation section of the Columbia Suicide Severity Rating Scale, if the ideation occurred in the past 6 months, or “yes” on any item of the Suicidal Behavior section, except for the “Non-suicidal Self Injurious Behavior” if this behavior occurred in the past 2 years

### Pain diary

Patients will keep a diary in which they will record their daily average pain score as assessed on the NRS (0–10) from the baseline week before treatment, throughout the treatment period and for another 3 weeks. The diary will be electronic (or, in some cases, on paper). The patients will also record their daily intake of study medication during the treatment period and their use of escape medication (number of tablets). The investigators will on a regular basis check that the diary is filled out during the treatment period, and daily during the first and last weeks to avoid missing data.

### Randomization

Randomization to the two treatments is done after the baseline period, by the pharmacy, using a computer-generated randomization list using block sizes unknown to the investigators. The patients will be stratified into two groups: patients with and without the irritable nociceptor phenotype. Allocation concealment will be ensured, as the randomization code will not be released until after the phenotype has been established and patients are randomized consecutively. The code for randomization will be stored in the pharmacy until the study is completed. Both sites receive for each randomization code a sealed envelope with information on the treatment given. The code envelope is only unsealed/opened in emergency cases if the safety of the patient requires knowledge of the randomization code.

### Compliance

In the pain diary, patients are required to record their intake of study medication morning and evening. Residual capsules shall be returned at the end of the treatment period (visit 3). The number is compared with the daily records of consumption of capsules in the dairy and the number of returned capsules is also recorded on the case report form. Furthermore, patients are contacted via telephone at least once during the treatment period and interviewed in a standardized manner to ensure compliance.

## Outcomes

### Primary outcome

- The difference in the mean value of the patients’ daily ratings of average pain intensity in the baseline week and the last week during treatment as experienced during the past 24 h rated on an 11-point numeric rating scale (NRS; 0 = no pain, 10 = worst possible pain)

### Secondary outcomes

- Pain relief (complete, good, moderate, mild, none, worse pain) (visit 3)
- Use (average numbers of tablets and number of subjects taking any dose) of escape medication (paracetamol) during the treatment period

### Tertiary outcomes

- The Patient Global Impression of Change (PCIG) measures the patients’ overall change (all aspects of general health) from baseline on a 7-point scale (very much improved, much improved, minimally improved, no change, minimally worse, much worse, very much worse) (visit 3)
- Pain impact on activities, sleep, and mood (NRS 0–10, from no impact to worst impact possible) (visit 2 and 3)
- Mean values of the daily pain ratings for the other 11 weeks
- Presence of 30% and 50% reduction of pain (from pain diary, baseline vs. last week of treatment)
- Symptoms of depression and anxiety and sleep disturbance assessed using the Patient-Reported Outcome Measurement Information System (PROMIS), the PROMIS questionnaire asks about symptoms experienced during the previous 7 days with a frequency or severity grading of symptoms; the scores are converted into PROMIS *T* scores, which are standardized relative to an American/US reference population, and to categories of impairment (normal and mild, moderate, and severe impairment) [16] (visits 2 and 3)
- The intensity of pain symptoms assessed by the Neuropathic Pain Symptom Inventory (NPSI) [7] (visits 2 and 3)
- Mechanical allodynia is assessed by brushing a soft brush (Somedic) twice with a speed of 1–2 cm/s and cold allodynia is assessed twice by a 20 °C cold thermal roller (Somedic); pinprick hyperalgesia is assessed using a pinprick stimulator as the difference in pain score (two stimulations at a control and at the pain side); pain is rated on the NRS (0–10) (visits 2 and 3)
- Hyperpathia assessed by repetitive mechanical pinprick stimulation at a rate of 2 Hz for 60 s and pain on the NRS (0–10) at 10-s intervals until the evoked pain has ceased [17] (visits 2 and 3)
- Nerve excitability testing is performed on the (nonaffected) wrist (visits 2 and 3)

### Other outcomes

- Adverse events assessed by open-ended questions
- Suicidal ideation and behavior assessed using the Columbia Suicide Severity Rating Scale [18]
- Assessment of blinding of trial; at visit 3, the assessment from the patient and investigator is recorded in the CRF, whether they think the patient received an active treatment or placebo, or do not know, and on what reason this is based (side effect, effect on pain, or something else) (visits 2 and 3)
- Assessment of patients’ expectations for the study drug (visit 1)
- A qualitative assessment of any difference in outcome based on the pain intensity ratings in the pain diary, the PGIC, and pain relief scores (visit 3)
- Blood samples will be taken for a biobank, and in a subgroup of patients treated with lacosamide we will perform genetic analyses of voltage-gated sodium channels, β-subunit 1–4, and Collapsin Response Mediated protein 2.

### Data management plan

Case report forms (CRFs) for each subject screened and enrolled in this study will be completed directly in the Research Electronic Data Capture (REDCap) database to the extent possible without the use of a paper CRF. REDCap is hosted by Aarhus University. Source documents include medical records, pain diaries, and CRFs (paper or REDCap (eCRF)). The study personnel at each site will be trained in the study procedures. All investigators will have access to the final trial data set. After the study, anonymized data will be available in a data repository and will be available upon request subject to written agreement with a department head.

### Statistics

With a minimally relevant between-phenotype group difference in total pain reduction of 1.25 NRS points, a standard deviation of 1.6 within phenotype groups [5], 80% power, and a 5% risk of type I error, the sample size estimate is 27 + 27 patients for the primary objective. With a minimally relevant treatment versus placebo difference in total pain reduction of 1.5 NRS points, 80% power, and a 5% risk of type I error, the sample size estimate is 30 + 15 patients using a treatment:placebo ratio of 2:1 for the supportive objective. With an expected dropout rate of 1/6 (data available for intention-to-treat (ITT) analysis), the recruitment stops when 54 patients in each phenotype group have been randomized to lacosamide and placebo in a 2:1 ratio. Thus, we expect to randomize 108 patients. For the explorative outcome in case there is no phenotype difference, 72 patients randomized to lacosamide and 36 patients randomized to placebo in the total population in an ITT population will give > 90% power to find a minimally relevant treatment versus placebo difference in total pain reduction of 1.25 NRS points and 85% power to find a minimally relevant treatment versus placebo difference in total pain reduction of 1.0 NRS points.

Statistical analysis of the primary outcome will be performed by *t* test and of the secondary outcomes by the Mann–Whitney *U* test. Nondichotomous tertiary outcomes will be performed by *t* test or Mann–Whitney *U* test, where applicable. Since we do not expect differences in baseline between the two phenotypes [5], a major impact of baseline pain intensity on the outcome, or a center effect, we do not plan to include these as covariates in the analyses.

For the primary outcome, the delta values from the average pain intensity in the baseline week to the last treatment week (last 7 days) will be used. Response rates and other dichotomous data are analyzed using Fisher’s exact test. For the primary (and supportive) objective, we are interested in the mechanistic aspects and in understanding whether the sensory phenotype “irritable nociceptor” is a prognostic biomarker for a sodium channel blocker in therapeutic doses. Therefore, the primary analysis for the primary objective is the PP population. Missing data will not be replaced. The PP population comprises those patients who complete at least 2 weeks on stable medication with at least 100 mg bid. Thus, if patients who fulfil the PP definition stop the treatment before the 12th week, the last seven pain scores on stable medication will be used for the primary analysis, and they will be invited for an additional visit identical to visit 3. All patients who have taken at least one study capsule will be encouraged to stay in the study, complete the diary, and come for a visit after 12 weeks.

As a secondary analysis, the ITT population will be used. The ITT analysis will also be used for the explorative outcome in the whole population if there is no phenotype difference. Given a substantial dose-dependent withdrawal rate due to adverse events, the EMA suggests a conservative responder analysis and that noncompleters are defined as nonresponders [19]. Therefore, the baseline observation carried forward (BOCF) in the ITT population (all patients randomized) will be the primary imputation method, and the last observation carried forward (LOCF) will be done as a secondary imputation method. Patients will be asked to complete the pain diaries despite their withdrawal from trial medication to minimize the need for imputation.

Significance is considered at the 5% level. If there are changes to the original statistical plan, the type of change and the date of change will be documented, and the document will be signed by the sponsor.

### Safety

Patients will record any adverse events in the pain diary and will be interviewed at each telephone call and study visit with open questions. The type of event, times of onset and termination, severity, and relationship with the treatment drug will be recorded.

### Publication

Regardless of the outcome, the results (including positive, negative, and inconclusive results) of the trial will be published in a recognized international journal. ICMJE guidelines for authorship will be followed.

## Discussion

The main aim of this study is to assess the concept of stratification based on pain phenotyping in neuropathic pain. We aim to assess whether we can reproduce results from a previous study showing a better effect of the sodium channel blocker oxarbazepine in patients with neuropathic pain and the so-called irritable nociceptor phenotype [5]. Lacosamide is a sodium channel blocker with a different profile enhancing the slow-inactivation of voltage-dependent sodium channels [8]. The strength of the study is that the primary purpose is to compare the effect in two groups of patients with different sensory pain phenotypes and thus possible different underlying pain mechanisms and blinding of persons involved in the study to the pain phenotype. There are several limitations. An unrealistically high number of patients would be required to power the study to show a difference in the drug–placebo differential between the two groups of patients, so the study will need to rely on supportive evidence. A thorough analysis of previous studies would have been advantageous to assess whether the best statistical plan should be a regression analysis including, for example, baseline pain intensity, center, and escape medication. There is a possibility of discontinuation due to lacosamide’s potential side effects and a risk of unblinding due to side effects. In addition, we do not know the strength and reproducibility of the phenotype classification as only one sensory testing will be performed, and the classification into IN and NIN can be considered an arbitrary dichotomy of continuous measurements [20].

## Trial status

At the time of first submission, the trial had not enrolled any patients. Recruitment started February 2019 and is expected to continue until the middle of 2021.

## Additional files

Additional file 1: Checklist SPIRIT. (DOC 121 kb)

Additional file 2: Informed consent. (PDF 460 kb)

## Data Availability

Not applicable.

## Abbreviations

bid: *bis in die* (twice daily)
CPM: Conditioned pain modulation
CRF: Case report form
IN: Irritable nociceptor
ITT: Intention to treat
NIN: Non-irritable nociceptor
NPSI: Neuropathic Pain Symptom Inventory
NRS: Numeric rating scale
PGIC: Patient Global Impression of Change
PROMIS: Patient Reported Outcome Measurement Information System
QST: Quantitative sensory testing
RCT: Randomized controlled trial
REDCap: Research electronic data capture
