# Supplementary material for: Effect of lacosamide in peripheral neuropathic pain: study protocol for a randomized, placebo-controlled, phenotype-stratified trial
Source: Trials. 2019 Oct 11;20:588. doi: 10.1186/s13063-019-3695-7 (PMC6788106; doi:10.1186/s13063-019-3695-7)
Supplement: Supplementary file 2 — Informed consent. (PDF 460 kb) [file 13063_2019_3695_MOESM2_ESM.pdf]

Informeret samtykke til deltagelse i et sundhedsvidenskabeligt forskningsprojekt.

Forskningsprojektets titel: Effekten af lacosamid på perifere neuropatiske smerter: Et randomiseret, dobbelt-blindet, placebo-kontrolleret, fænotypestratificeret studie

Erklæring fra forsøgspersonen:

Jeg har fået skriftlig og mundtlig information og jeg ved nok om formål, metode, fordele og ulemper til at sige ja til at deltage.

Jeg ved, at det er frivilligt at deltage, og at jeg altid kan trække mit samtykke tilbage uden at miste mine nuværende eller fremtidige rettigheder til behandling.

Jeg giver samtykke til, at deltage i forskningsprojektet, og har fået en kopi af dette samtykkeark samt en

kopi af den skriftlige information om projektet til eget brug.

Forsøgspersonens navn: ____

Dato: _______________ Underskrift: ____

Ønsker du at blive informeret om forskningsprojektets resultat samt eventuelle konsekvenser for dig?:

Ja _____ (sæt x) Nej _____ (sæt x)

Erklæring fra den, der afgiver information:

Jeg erklærer, at forsøgspersonen har modtaget mundtlig og skriftlig information om forsøget.

Efter min overbevisning er der givet tilstrækkelig information til, at der kan træffes beslutning om deltagelse i forsøget.

Navnet på den, der har afgivet information: _______________________________________

Dato: _______________ Underskrift: ____

Projektidentifikation: **EudraCT number 2018-003110-40, protocol number LACOSAMIDE-2018**
